# Feminization of the health workforce in China: exploring gendered composition from 2002 to 2020

**DOI:** 10.1186/s12960-024-00898-w

**Published:** 2024-02-19

**Authors:** Mingyue Li, Joanna Raven, Xiaoyun Liu

**Affiliations:** 1 Department of Health Policy and Management, School of Public Health, Peking University, Beijing, People’s Republic of China; 2 China Center for Health Development Studies, Peking University, 38 Xueyuan Road, Haidian District, Beijing, 100191 People’s Republic of China; 3 Division of Primary Care and Population Health, Department of Medicine, School of Medicine, Stanford University, Stanford, California USA; 4 Department of Public Health, Liverpool School of Tropical Medicine, Liverpool, UK

**Keywords:** Gendered composition, Health workforce, China

## Abstract

**Background:**

Feminization of health workforce has been globally documented, but it has not been investigated in China. This study aims to analyze changes in the gendered composition of health workforce and explore the trend in different types of health workforce, health organizations and majors within China’s health system.

**Methods:**

The data were collected from China Health Statistical Yearbook from 2002 to 2020. We focused on health professionals including doctors, nurses, and pharmacists in health organizations. Trend analysis was employed to examine the change in the ratio of female health workforce over 18 years. The estimated average annual percent change (AAPC) was estimated, and the reciprocals of variances for the female ratios were used as weights.

**Results:**

In China, health professionals increased from 4.7 million in 2002 to 10.68 million in 2020. Health professionals per 1000 population increased from 3.41 in 2002 to 7.57 in 2020. The ratio of female health professionals significantly increased from 63.85% in 2002 to 72.4% in 2020 (AAPC = 1.04%, 95% CI 0.96–1.11%, P < 0.001). Female doctors and pharmacists increased 4.7 and 7.9 percentage points from 2002 to 2020. Female health workers at township health centers, village clinics, centers for disease control and prevention had higher annual increase rate (AAPC = 1.67%, 2.25% and 1.33%, respectively) than those at hospital (0.70%) and community health center (0.5%). Female doctors in traditional Chinese medicine, dentistry and public health had higher annual increase rate (AAPC = 1.82%, 1.53% and 1.91%, respectively) than female clinical doctor (0.64%).

**Conclusions:**

More women are participating in the healthcare sector in China. However, socially lower-ranked positions have been feminizing faster, which could be due to the inherent and structural gender norms restricting women’s career. More collective and comprehensive system-level actions will be needed to foster a gender-equitable environment for health workforce at all levels.

## Background

Gender equality is a fundamental human right and is a necessary foundation to achieve a peaceful and prosperous world [1]. Women’s participation in the labor market is stalling in the world [2]. By 2022, women labor force participation is just over 50% compared to 80% for men [3]. While China’s women labor force participation is still higher than the global average, it has decreased from 79.3% in 1990 to 68.6% in 2019. Women are constantly under-represented in sectors like finance, law, science and technology, and government. In 2022, only 13.8% of all board of directors are women in China [4].

The health care sector has become the fastest-growing employment sector for women globally. In 2022, women make up over 70% of the health workforce compared with 39.5% in 2013, most represented at the low-paid tiers among nurses and community health workers [5, 6]. Women health workers deliver services to around 5 billion people in the world and contribute $3 trillion to global health annually [7]. In addition, more women are entering health care education [8, 9], and have outnumbered men in medical schools in countries like Germany [10], the United States [11], Japan [12], and African countries [13]. Identifying the trends and dynamics of the gendered composition in health workforce is important to learn restrictive gender norms and inequalities in health systems. It also provides basic evidence for supportive human resource management policies, practices that are required to advance Universal Health Coverage and a sustainable health system.

Health system is not gender neutral. Relational theory understands gender as a multidimensional concept that embraces complex economic, power and symbolic relations and operates at all levels [14]. While gender is a fundamental stratifier of health workforce, it will inevitably intersect interests, power relations and values. However, feminization of health workforce has not received adequate attention in China, a country with a 1.4 billion population and 688.4 million women. Studies on China’s health workforce have not explored the gendered composition or its change. For example, Tang mentioned the feminization of physician workforce supply but did not explore its degree or its change [15].

It is important to understand China’s health system before analyzing the feminization of its health workforce. China’s health system has a hierarchical structure, which consists of a public health system and a medical care system. The public health system is mainly composed of centers for disease control and prevention (CDCs), and maternal and child health hospitals. The medical care system is composed of primary health care facilities (township health centers [THCs], community health centers [CHCs] and village clinics [VCs]), and secondary and tertiary hospitals [16]. THCs and VCs are located in rural areas, while CHCs are located in urban areas. There are significant disparities between the distribution and qualifications of the health workforce in urban and rural areas, particularly for primary health workers [17, 18]. In Chinese health worker nomenclature**,** clinical medicine positions in secondary and tertiary hospitals are seen as more prestigious than roles in traditional Chinese medicine and public health [19].

To address the knowledge gap on gendered characteristics of China’s health workforce, we used national data from 2002 to 2020 to analyze changes in the gendered composition of health workforce in various types, health organizations and majors. Our findings could provide clear evidence on the shift and distribution of gender in China’s health workforce, and trends of feminization in different social and economic positions. This study could add strong evidence from China to the global gender transition of health workforce and identify evidence gaps for future research.

## Methods

### Data sources

The data were collected from China Health Statistical Yearbook from 2002 to 2020. The National Health Commission (formerly the Ministry of health) of China collected and published the statistics annually. The Yearbook reports statistics that reflect the development of the health resources, health expenditure, health services utilization, population health status, diseases control and prevention, health insurance and other related topics in China. The Yearbook is the official data source for understanding China’s health care; it contains statistics for 31 provinces, autonomous regions, and municipal cities. Data on both public and private health organizations are included. The Yearbook provides information on health workforce data, such as the number of health workers across various types, regions, and health organizations. In 2002, the Yearbook initially published sex-disaggregated data for health workers. The Yearbook did not publish sex-disaggregated data for the years 2003–2004 and 2006–2008. Since 2009, the Yearbook has been publishing sex-disaggregated data for various types of health workers and health organizations. In this paper, we focused on the ratios of male to female health workers across various types and organizations.

### Definitions for health workers and other health cadres

China’s health worker nomenclature includes health professionals and non-professionals. Doctors, nurses, and pharmacists are among the main healthcare providers as health professionals. Management staff and other technicians are the main non-professionals [16]. China’s village doctors are comparable to community health workers in the global context [20]. Prior to 1985, China’s village doctors were referred to as barefoot doctors. People who had basic education (secondary school) received short-term training in local hospitals before beginning to provide basic health care in communities. It should be noted the majority of barefoot doctors were men because most women at the time lacked formal schooling. This paper focuses on health professionals, specifically doctors, nurses, and pharmacists (Table 1).

**Table 1:** Categories and descriptions of various types of cadres

Cadre titles	Description
Health workers	Refer to staff members who work in hospitals, PHC facilities (VC, THC, CHC), public health facilities (primarily CDC), and other health care facilities *In China’s context, the definition of ‘health worker’ is narrower compared to the WHO classification. The WHO 2019 international classification of health workers included five groupings: health professionals, health associate professionals, personal care workers in health services, health management and support personnel, and other health service providers not elsewhere classified. The WHO definitions for ‘health workers’ cover a broad range of occupations, such as health economists*
Health professionals	Include doctors, nurses, pharmacists, laboratory technicians, radiology technicians, intern doctors, and other health professionals not elsewhere classified. Those who engage in management, such as directors of hospitals, are not included in this category
Doctors	Refer to a type of health professionals who have passed a licensing examination and are registered at a health authority of county-level or higher-level. Doctors include licensed doctors (medical graduates who graduated with at least a bachelor’ degree) and licensed assistant doctors (medical graduates who graduated from 3-year tertiary medical education programs with an associate degree or 2-year secondary education programs with a diploma)
Nurses	Refer to a type of health professionals who have acquired nursing qualifications with at least a 3-year tertiary nursing education and are engaged in nursing activities
Pharmacists	Refer to a type of health professionals who are responsible for providing medication and pharmaceutical services

### Data analysis

We first illustrated the trends in the overall numbers of China’s health workforce overtime. Next, we performed a descriptive analysis of the gender distribution of health workforce from 2022. Considering China’s hierarchical health system structure, we further analyzed the changes in various types of health workers and health workers at various levels of health organizations. The estimated average annual percent change (AAPC) was estimated using the following model. The AAPC is ${({\text{ratio}}}_{i+1}-{{\text{ratio}}}_{i})/{{\text{ratio}}}_{i}={\text{exp}}(\beta )-1.$ The 95% confidence interval and corresponding p value were reported. Weighted least squares was used for estimation. Weights based on the reciprocals of variances for the female ratios were used to account for the variability in the ratios [21, 22]:

$${\text{log}}\left({{\text{ratio}}}_{i}\right)={\beta }_{0}+\beta {{\text{year}}}_{i}+\epsilon , i=1, \cdot \cdot \cdot , 14$$

Microsoft Excel 2019 and Stata 16.0 (Stata Corp LP, College Station, TX, USA) were used to perform all analyses.

## Results

### Changes in quantity of health workforce and health professionals

**Fig. 1:**
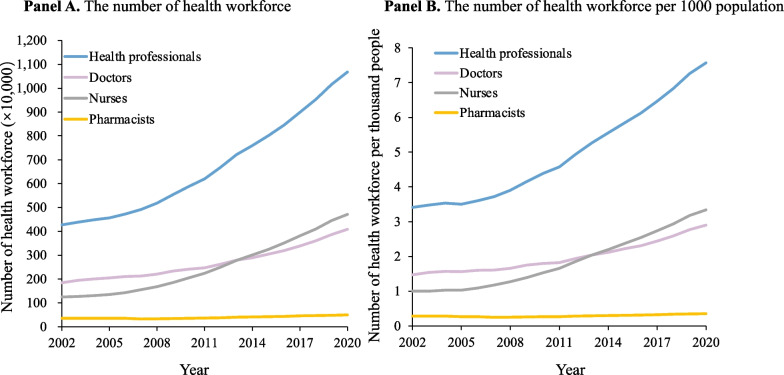
Trend of the health workforce in China (2002–2020)

The health workforce in China has grown steadily since 2002. The number of health professionals increased from 4.70 million in 2002 to 10.68 million in 2020 (Fig. 1). Doctors increased from 1.84 million to 4.08 million, and nurses increased from 1.25 million to 4.71 million (Fig. 1A). In addition, there was an increase in the number of the total health workforce per 1000 population (3.41 in 2002 to 7.57 in 2020). The suggested threshold in Sustainable Development Goals is 4.45 doctors, nurses and midwives per 1000 population [23]. Nurses (1.00 in 2002 to 3.34 in 2020) increased faster than doctors (1.47 in 2002 to 2.90 in 2020) (Fig. 1B).

### Gendered composition of health professionals

**Fig. 2:**
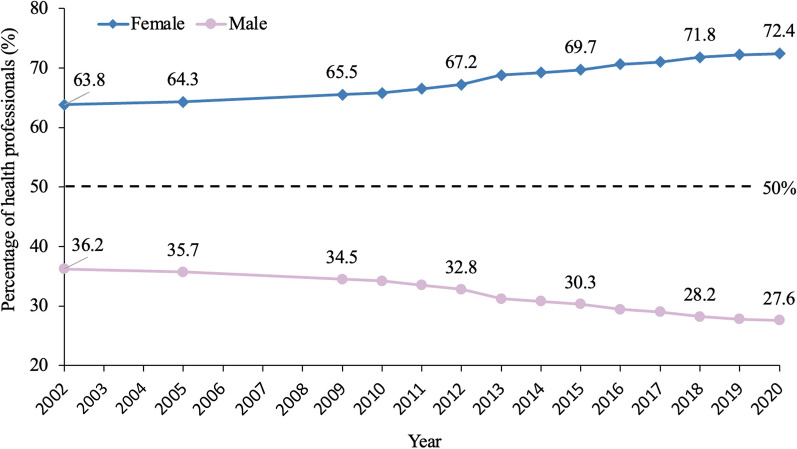
Trend of gendered composition of health professionals in China (2002–2020). Sex-disaggregated data in 2003–2004 and 2006–2008 were not published

Female ratio of health professionals increased significantly from 63.8% in 2002 to 72.4% in 2020 (AAPC = 1.04%, 95% CI 0.96–1.11%, P < 0.001) (Fig. 2).

### Gendered composition of different types of health workers

**Fig. 3:**
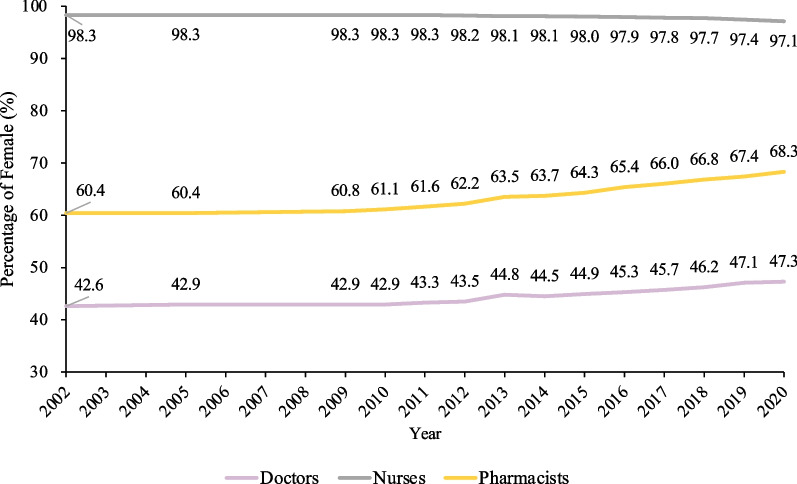
Trend of female composition in different health workforce cadres in China from 2002 to 2020. Sex-disaggregated data in 2003–2004 and 2006–2008 were not published

Female doctors increased from 42.6% in 2002 to 47.3% in 2020. Female pharmacists also showed a 7.9% increase from 60.4% to 68.3%. The gendered composition was balanced in doctors (47.3% in 2020) and pharmacists (68.3% in 2020). Nursing workforce were predominantly women (97.1% were women in 2020), with a slight decrease of 1.2% (Fig. 3).

### Gendered composition of health workforce by health organizations

At VCs and THCs, the ratio of female health workforce increased significantly by 2.25% (95% CI 1.90–2.60%, P < 0.001) and 1.67% (95% CI 1.56–1.78%, P < 0.001) per year, respectively. At CDCs, the ratio of female health workforce increased by 1.33% every year (95% CI 1.18–1.48%, P < 0.001). Compared to VCs and THCs with their locations in rural areas, hospitals and CHCs with their locations in urban areas have seen a lower increase in the ratios of female health workers.

### Gendered composition of doctors by majors

The distribution of genders among doctors was balanced across majors. In all four sub-groups—clinical medicine, traditional Chinese medicine, dentistry, and public health—there has been a significant increase in the ratios of female doctors. For clinical medicine, there was an average 0.64% increase in female doctors each year (95% CI 0.52–0.76%, *P* < 0.001). The AAPC was the highest in public health (1.91%) and ranked second in Traditional Chinese Medicine (1.82%) (Table 2). In China, positions in public health and Traditional Chinese Medicine are perceived as ‘socially lower-ranked’ in comparison to positions in clinical medicine as these positions [24, 25]. These positions are lower paid and less well known to the public compared to clinical medicine.

**Table 2:** Ratio of female health workforce in different types of health organizations in China (2002–2020)

Year	Medical system				Public health system	
	Hospital	Community health center	Township health center	Village clinic	Maternal and child health hospital	Center for Disease Control and Prevention
2002	68.5	73.9	52.2	–	82.4	–
2005	68.3	71.8	53.1	–	82.3	48.8
2009	69.7	70.9	55.4	–	83.1	51.5
2010	70.0	71.2	55.7	–	83.2	51.8
2011	70.5	70.2	56.1	–	83.4	52.0
2012	71.0	70.4	56.7	–	83.6	52.2
2013	72.2	71.8	58.6	29.2	84.2	53.7
2014	72.4	72.0	59.0	29.6	84.3	53.9
2015	72.8	72.7	59.8	29.9	84.4	54.5
2016	73.3	74.2	61.3	30.5	84.7	55.7
2017	73.6	74.9	62.3	31.1	84.9	56.3
2018	74.1	75.2	63.4	32.5	85.2	57.1
2019	74.3	75.2	64.3	32.8	85.2	57.7
2020	74.4	76.2	64.8	34.1	85.1	58.2
AAPC	0.70%	0.50%	1.67%	2.25%	0.28%	1.33%
95% CI	(0.63%, 0.77%)	(0.24%, 0.76%)	(1.56%, 1.78%)	(1.90%, 2.60%)	(0.25%, 0.31%)	(1.18%, 1.48%)
*P* value	< 0.001	0.003	< 0.001	< 0.001	< 0.001	< 0.001

AAPC, estimated average annual percent change

### Gendered composition of health workforce by majors and organizations

Table 4 presents the ratio of various types of female health workforce by levels of health organizations. The gendered composition was balanced in doctors across organizations, except doctors in MCHs and VCs. Women made up the majority of pharmacists and nurses across organizations. The female doctor ratio in the medical system (hospitals, THCs, and VCs) all showed a significant increasing trend. The increase was greater in THCs (AAPC = 1.35%, P < 0.001) and VCs (AAPC = 1.59%, P = 0.002) in rural areas compared with other organizations (AAPC = 0.63%, P < 0.001), while female doctors in CHCs in urban areas remained unchanged during the 18 years (AAPC = 0.15%, P = 0.670).

As for health organizations in the public health system, approximately 3/4 of doctors were women. Female doctors increased significantly from 41.0% in 2005 to 48.7% in 2020 in CDC (AAPC = 1.49%, P < 0.001).

Women predominate nurses. From 2002 to 2020, there was a marginal rise in the ratios of male nurses in all organizations (for example, women decreased 0.13% on average annually in the hospitals P < 0.001), with the exception of THCs, where the ratio of nurses increased slightly (AAPC = 0.07%, P < 0.001) (Tables 3, 4).

**Table 3:** Ratio of female doctors in different majors in China (2002–2020)

Year	Total	Clinical medicine	Traditional Chinese medicine	Dentistry	Public health
2002	42.6	44.7	32.5	43.1	38.7
2005	42.9	44.7	33.5	43.5	39.5
2009	42.9	44.4	33.2	43.7	41.3
2010	42.9	44.4	33.0	44.0	41.7
2011	43.3	44.6	33.5	44.6	42.2
2012	43.5	44.7	33.8	45.0	42.2
2013	44.8	46.0	35.2	46.4	44.1
2014	44.5	46.0	35.3	46.5	44.0
2015	44.9	46.3	35.9	47.1	44.7
2016	45.3	46.6	37.0	48.2	46.4
2017	45.7	46.8	37.6	48.9	47.0
2018	46.2	47.2	38.9	50.1	48.0
2019	47.1	47.7	40.1	51.3	49.0
2020	47.6	48.1	41.6	52.6	50.2
AAPC	0.86%	0.64%	1.82%	1.53%	1.91%
95% CI	(0.75%, 0.97%)	(0.52%, 0.76%)	(1.53%, 2.11%)	(1.37%, 1.69%)	(1.77%, 2.05%)
*P* value	< 0.001	< 0.001	< 0.001	< 0.001	< 0.001

AAPC, estimated average annual percent change

**Table 4:** Ratio of different types of female health workforce by levels of health organizations in China (2002–2020)

Year		2002	2005	2009	2010	2011	2012	2013	2014	2015	2016	2017	2018	2019	2020	AAPC	95% CI	*P* value
Doctors																		
Medical system	Hospital	43.4	43.2	42.5	42.5	42.8	42.9	43.8	43.4	43.8	44.5	44.8	45.3	46.3	46.5	0.63%	(0.44%, 0.82%)	< 0.001
	CHC	62.6	56.7	53.4	53.2	52.2	52.4	53.6	53.6	54.3	55.8	56.4	56.9	57.8	58.2	0.15%	(− 0.53%, 0.83%)	0.670
	THC^#^	35.1	35.9	36.6	36.7	36.6	37.1	37.9	37.2	37.5	38.8	39.6	40.6	42.2	42.7	1.35%	(1.08%, 1.62%)	< 0.001
	VC^#^	–	–	–	–	–	–	31.8	29.7	29.9	30.5	30.9	32.3	32.5	34.8	1.59%	(0.42%, 2.76%)	0.037
Public health system	MCH	76.5	76.3	74.5	74.5	74.2	73.8	73.9	74.1	73.8	74.1	74.1	74.1	73.7	73.6	− 0.21%	(0.32%, − 0.10%)	0.002
	CDC	–	41.0	41.5	41.7	42.1	42.4	44.0	43.8	44.3	45.5	46.2	47.0	48.2	48.7	1.49%	(1.35%, 1.63%)	< 0.001
Pharmacists																		
Medical system	Hospital	63.4	63.3	63.3	63.4	63.7	64.0	64.9	65.1	65.6	66.5	66.9	67.5	68.0	68.3	0.66%	(0.57%, 0.75%)	< 0.001
	CHC	70.6	67.4	68.3	68.5	68.5	68.7	69.7	69.8	70.7	72.1	73.1	73.8	74.9	75.5	0.78%	(0.54%, 1.02%)	< 0.001
	THC^#^	50.7	50.6	50.2	50.7	51.2	52.3	54.3	54.6	55.6	57.5	58.7	60.1	61.5	62.3	1.83%	(1.59%, 2.07%)	< 0.001
Public health system	MCH	74.6	74.2	73.1	73.1	73.2	73.0	73.5	73.5	73.4	74.1	74.4	74.7	74.7	74.9	0.11%	(0, 0.22%)	0.063
	CDC	–	66.8	63.3	63.3	63.9	63.8	63.9	63.6	63.9	64.7	64.4	64.8	64.5	64.6	0.02%	(− 0.19%, 0.23%)	0.851
Nurses																		
Medical system	Hospital	98.4	98.4	98.3	98.2	98.2	98.1	97.9	97.9	97.8	97.6	97.5	97.3	97.0	96.6	− 0.13%	(0.15%, − 0.11%)	< 0.001
	CHC	99.6	99.4	99.3	99.3	99.3	99.3	99.4	99.4	99.4	99.4	99.4	99.3	99.3	99.2	− 0.01%	(0.02%, 0)	0.050
	THC^#^	97.5	97.6	98.1	98.2	98.3	98.3	98.4	98.4	98.4	98.5	98.6	98.5	98.6	98.5	0.07%	(0.05%, 0.09%)	< 0.001
	VC^#^	–	–	–	–	–	–	94.5	94.9	95.1	74.9	25.6	38.7	58.8	70.0	− 9.37%	(21.57%, 2.83%)	0.202
Public health system	MCH	99.1	99.1	99.2	99.2	99.2	99.2	99.2	99.2	99.2	99.3	99.2	99.2	99.0	98.9	− 0.01%	(0.02%, 0)	0.315

AAPC, estimated average annual percent change; THC, township health center; CHC, community health center; MCH, maternal and child health hospital; CDC, Center for Disease Control and Prevention; ^#^based in rural areas

## Discussion

This study examined the gendered composition of China’s health workforce and analyzed the trend for various types and professions using the national health statistics. To our knowledge, this study is the first to investigate the feminization of the Chinese health workforce. Our study highlights the following findings. First, the health workforce in China is pre-dominantly composed of women, with their proportion increasing from 63.8% in 2002 to 72.4% in 2020. Second, the rural health workforce is feminizing at a faster speed than urban. Finally, socially lower-ranked health workers are feminizing at a faster speed compared to those in more prominent and authoritative professions in China.

Our findings showed a significant 1.04% AAPC increase in female health professionals, which is consistent with existing literature. The movement of women into healthcare professions has been extensively documented in global countries [26], particularly in low- and middle-income countries like Latin America [27], Africa [13], Sultanate of Oman [28], Bangladesh [29]. In Bangladesh, the ratios of female doctors increased from 47.9% in 2006 to 52.5% in 2015 [29]. Similarly, in Canada and the United States, the ratios of female physicians and surgeons rose from under 5% in 1930 to over 30% in 2008 [26]. Shannon et al. estimated that the annual increase in women health professionals was 4.5% in low–middle-income countries, 5.8% in upper middle-income countries, and 1.1% in high-income countries [30].

The feminization of health workforce has sparked extensive discussion regarding its underlying factors, causes, nature, and potential impact [6]. Research has documented sex differences in specialty choosing [31], quality of care provided [32, 33], and research productivity [34]. Concerns also exist regarding workforce supply and provision of health services [35], work patterns [36, 37], imbalances in specialties [38], social perception of the healthcare profession [26], and equity [6, 39, 40] because of this disproportionate feminization. However, in many contexts, sex differences reflect restrictive gender norms and inequalities. For example, research from the United States found that women are discouraged from pursuing certain specialties such as surgery, during early medical training [41].

The feminization of the health workforce in China closely follows its fast economic growth and the resulting social transition, including changes in the meso environment [42]. Since the establishment of the People’s Republic of China, women’s social status has significantly improved. Multiple laws and regulations have been implemented to safeguard women’s rights, most notably the Law on the Protection of Women’s Rights and Interests (2005). This law stipulates that women have equal rights with men “in all aspects of political, economic, cultural, social and family life”. Women now have more opportunities of for education and enrollment in healthcare colleges. China’s gross enrollment rate in higher education was 54.4% in 2020, marking a 27.9 percentage point increase from 2010. Women account for a larger proportion of undergraduates in regular HEIs (51%, 3.1 percentage points higher than 2010), postgraduates (50.9%, 0.1 percentage points higher than 2010) as well as undergraduates in adult HEIs (58%, 4.9 percentage points higher than 2010) [43]. Girls tend to favor medical schools in higher education because careers in medical fields are perceived as noble, secure and stable [44].

The shift of meso environment in health care may also contribute to feminization. First, the developing and expanding health care sector releases higher demand for health workforce. In China, a relatively well-developed health system has been established over the last century, leading to a higher demand for health workers [16]. Second, population aging and increasing non-communicable disease burden have further increased healthcare needs and demand for health workers [45]. Third, the COVID-19 pandemic has also led to increased health care needs and the demand for more health workers, but further research is needed to understand its long-term effects on women’s participation in health workforce. The COVID-19 will leave permanent scar on health labor market. Preliminary studies have found that women health workers experienced a higher risk of exposure and infection, as well as reduced leadership opportunities and increased mental strain [46]. These negative impacts may discourage women from participating in health workforce in the future.

There are complex underlying factors and causes for faster feminization in socially lower-ranked cadres in China. Medical students still adhere to traditional gender stereotypes when selecting specialties. For example, female students are more likely to choose socially lower-ranked specialties like internal medicine [47]. Women are often perceived as less skilled and are typically assigned traditional gender roles as caregivers, while men are given priority for higher-training positions [48]. Women also often bear a greater unpaid domestic burden and struggle to maintain a work-family balance [49]. Furthermore, lower positions leave women vulnerable to abuse, harassment and devaluation [5]. Despite workforce feminization, there are inherent power dynamics, with women occupying low-paid tiers in China. The feminization of certain medical specialties indicated the gender occupation hierarchy [6].

Rural health workforce is feminizing at a faster speed than urban. Besides macro-economic factors abovementioned, increasing job opportunities in cities attracted men to urban areas for more lucrative jobs, while leaving women to fill the vacancies in rural health organizations [48]. Besides, the implementation of China’s Equalization of Basic Public Health Services policy in 2009 has also contributed to the increased participation of women in rural health organizations. This policy aims to enhance UHC by strengthening public health services. As a result, health workers in VCs and THCs have less field work and more administrative desk work, such as establishing and maintaining health records for all citizens, health monitoring and regular follow-ups [50]. Jobs in these health facilities are considered as more stable and more suitable for women in China’s traditional gender norms.

Majors in socially lower-ranked positions, including traditional Chinese medicine and public health (mainly CDC), have been feminizing at a faster speed. The accelerated feminization in socially lower-ranked positions is a reflection of the restrictive gender occupation hierarchy and gender inequity. Women are selectively channeled into socially lower-ranked positions which point to differential career paths within the status hierarchy. Men concentrate in socially higher-ranked positions, which often entitles them to more administrative power over women-dominated positions, thereby reinforcing the gendered occupation hierarchy [51]. Our finding highlights the pressing need to improve gender equity at the health system level.

The feminization of the health workforce could be beneficial. Gender diversity is able to translate into an empowered health workforce and improved patient outcomes. Extensive evidence from various fields demonstrates that gender diversity leads to substantial gains in productivity, innovation, and employee retention [52]. Growing evidence shows the way female doctors practice leads to reduced mortality and better patient outcomes [32, 33]. Although gender differences exist in practice approaches, they are not inherent and unchangeable [53], because a gender-diverse environment has the potential to drive innovation and influence behaviors [54]. Except doctors in VC and MCH, doctors are now gender-balanced across majors and organizations, which will contribute to a gender-diverse environment. The health sector needs to invest more efforts to achieving gender diversity among pharmacists and nurses, who are still predominantly women, especially when 97.1% of the nursing workforce are female in 2020.

However, there are concerns regarding the feminization of certain sectors. When a sector has a high proportion of women, it may experience a decline in status [55, 56]. Professions that are predominated female tend to be considered as low status, while those dominated by men are often considered as high status. For example, nursing has long been dominated by women in China and is perceived as socially lower-ranked than doctors in the labor market. Similarly, in Russia and Estonia, medicine is a female-dominated profession and is also perceived as low-status. One hypothesis suggests that as certain sectors become less attractive to men, they tend to become feminized, with women stepping in to fill the resulting job vacancies [55]. The loss of prestige predates the feminization process. According to the relational theory, feminization can potentially reshape the original power relations, economic relations and symbolic relations within the health workforce, bringing about power negotiations in decision-making process, values and other aspects of health system [14].

Despite the increasing number of women becoming doctors, female doctors are still experiencing differential treatment [57, 58]. According to a cohort of medical graduates, female general practitioners in primary healthcare facilities had similar wage with their male classmates. However, female doctors working in hospitals earned less than their male classmates, and this gap widened over time [59, 60]. This study did not specifically analyze gender pay gap due to data limitations, while our findings highlighted the urgency of investigating the gender pay gap within the health workforce, especially as more women enter the field.

Our study has implications for policies in multiple ways. There is an urgent need to integrate gender equity in health workforce policies to support women and foster a gender-equitable environment. Effective gender-transformative policies should be tailored to the specific context, particularly in rural areas, public health, and traditional Chinese medicine. There is no strict optimized gender ratio of health workforce because gender equity is also shaped by other important intersectional factors, such as race, ethnicity, class, and geography [61]. Gender equity in health workforce is not just about achieving a certain gender ratio, but also about creating an inclusive environment where all employees feel valued and respected, regardless of their gender identity.

Our study has also highlighted some key evidence gaps. Future research should explore a better gender ratio to reflect gender equity in health workforce while accounting for intersectoral factors. Research should also examine the complex impact of feminization on health human resources supply. For example, could majors that were previously dominated by men experience a shortage in labor supply?

Gender transition should be considered as an important influencing factor in health workforce planning [13, 62]. On one hand, health workers continue to face differential responsibilities based on their socially ascribed gender roles, which may affect workforce supply on certain specialties. On the other hand, women health workers may adapt their working patterns in more supportive environment and communities, which may compensate for the social ascribed gender roles.

### Limitations

This study is subject to several limitations. First, the aggregate data did not allow for more detailed analysis, such as examining the differences in the female/male ratio among doctors specializing in surgery and internal medicine, gender differences in career development, and gender differences in economic situations between regions and provinces. Second, due to data limitations, we were not able to investigate gender pay equity. Studies from the United States have found an apparent gender pay gap among dentists [58, 63]. With feminization of health workforce underway, future research should further explore gender pay equity and quantify the extent to discrimination contributes to the gender pay gap in health care field. The health sector should start to increase their gender-responsiveness of work environment to prepare for the feminization of their workforce.

## Conclusion

More women are participating in the healthcare sector in China. Improvement in women’s social status, increased representation of women in higher education, and health care market expansion may have contributed to this transition. However, there are still challenges. The faster feminization in socially lower-ranked positions exposes the underlying gender disparity in the health system. Inherent and structural gender norms restrict women’s career. More collective and comprehensive system-level support will be needed to create a gender-equitable environment for health workforce. More research is needed to investigate barriers to women’s entry in fields that remain male-dominated and to explore the impact of more women in the health sector.

## Data Availability

The data sets generated and/or analyzed during the current study are publicly available from the China Health Statistical Yearbook.

## Abbreviations

AAPC: Estimated average annual percent change
THC: Township health center
CHC: Community health center
MCH: Maternal and child health hospital
CDC: Center for disease control and prevention
PHC: Primary health care
